# A mid-level explanation for the venetian blind effect

**DOI:** 10.3389/fpsyg.2013.00908

**Published:** 2013-12-03

**Authors:** Barbara J. Gillam, Susan G. Wardle

**Affiliations:** School of Psychology, University of New South WalesSydney, NSW, Australia

**Keywords:** venetian blind effect, stereoscopic slant, stereopsis, binocular vision, depth perception

**Figure 1:**
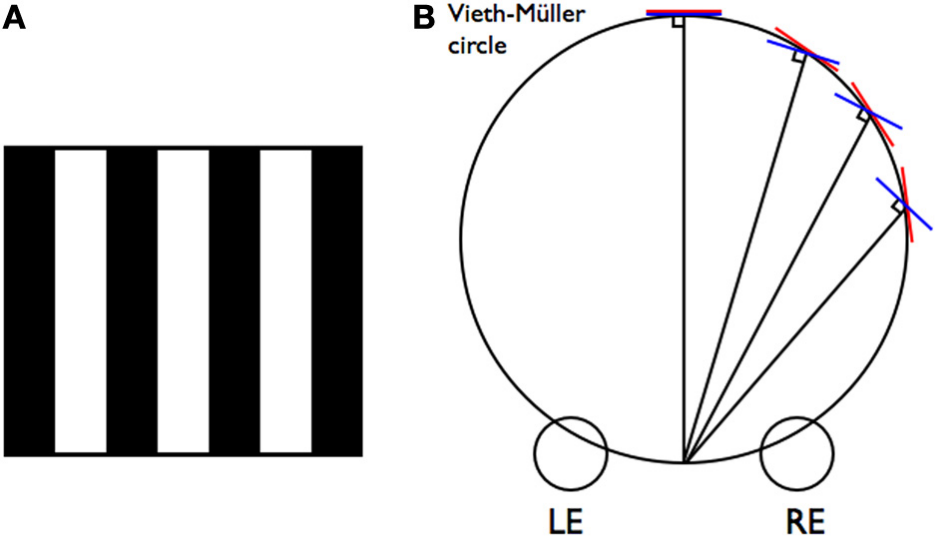
**(A)** Demonstration of the venetian blind effect. When viewed with a neutral density filter (or polarized sunglasses) covering one eye, the white bars appear to slant in the direction toward the covered eye. **(B)** The deviation between the horopter (red lines) tangent to the Vieth-Müller circle, and the normal (blue lines) which is at right angles to the line of sight, increases with increasing visual field eccentricity. In central vision the tangent and the normal coincide.

The term “venetian blind effect” was first coined by Cibis and Haber (1951) to describe a phenomenon in which a black and white vertical grating (Figure 1A) viewed with a neutral density filter over one eye, appears as a set of white slats, each one slanted around a central vertical axis appearing nearer on the side with the filter. A source of confusion is that Ogle (1962) renamed it “irradiation stereoscopy” and the term “venetian blind effect” has also been applied to another phenomenon. When gratings of different spatial frequency are presented to the two eyes, the pattern may break up into local slanted regions rather than slanting as a whole. This case has obvious horizontal disparity between the images and the question is how this pattern of disparities is organized under different combinations of spatial frequency. This is worthy of investigation. However, the original venetian blind phenomenon and the only one we discuss here, is much more mysterious and interesting because there is no horizontal disparity introduced by imposing a luminance or contrast difference between the two eyes' views.

The venetian blind effect has been almost universally attributed to early neurophysiological processes such as irradiation or blur (Münster, 1941; Cibis and Haber, 1951; Ogle, 1962; von Békésy, 1970). Although there is no disparity in the distal images presented to the left and right eyes, processes such as irradiation could introduce physical image differences at an early processing stage with a resulting horizontal disparity. The degree of slant perceived does increase with the magnitude of the luminance difference in the two eyes (Cibis and Haber, 1951). However, experimental work to date (which has been limited) does not support these theories (Filley et al., 2011; Dobias and Stine, 2012) and the effect remains a mystery. Filley et al. (2011) have recently proposed a model in which depth is assigned to monocular edges and modulated by the inter-ocular difference in intensity (see also Hetley and Stine, 2011). Here we take a different approach from previous work and propose a mid-level explanation for the venetian blind effect. Of course this does not rule out complementary physiological explanations.

There is another binocular phenomenon associated with introducing a very large luminance difference between the two eyes' images of a fusible binocular stimulus—“binocular luster.” Helmholtz (1910) attributes luster to the fact that only a surface with a bias to reflect light in a particular direction can produce different luminances in the two eyes. One eye can be “in the direction where the light is reflected while the other is not.” Only a lustrous object will reflect light in such a highly directional fashion. A dull object will reflect light equally in all directions. Hence an otherwise fusible surface with a luminance difference will appear lustrous. Helmholtz's theory of luster is an example of his ecological approach to stereopsis in marked contrast to Hering's sensory approach. Can we apply a similar ecological analysis to the venetian blind effect?

In the venetian blind effect each white strip appears slanted toward the eye with the filter (Figure 1A). Under what set of binocular stimulus conditions would this occur? Frontal plane surfaces in front of the eyes would reflect approximately equal amounts of light in the directions of the two eyes. However, a slanted surface would reflect more light into one eye than the other. The eye that is more normal to the slanted surface would receive more light than the eye that is more obliquely positioned. Hence relative luminance in the two eyes could be regarded as a stereoscopic slant cue, indicating that the surface is slanted away on the brighter side, i.e., the side without the filter. The greater the difference in luminance, the greater the slant would have to be to account for it. (The luminance differences in the venetian blind effect are never as great as the extreme differences that give rise to luster). Only the white slats would have this property. As the alternate slats are black they would not register the effect of the filter, hence the slant is induced only in the white slats. The black slats are, however, sometimes seen slanted in the opposite direction. This could simply be extrapolation from the perceived depth of the edges they share with the white slats.

This situation could be regarded as a conflict because the slats have no binocular disparity so should not appear slanted even though the luminance information is consistent with slant. Dobias and Stine (2012) note the contrast disparity in the venetian blind effect and suggest that it could possibly be used for slant perception at distances where horizontal contour disparity is no longer useful, although Allison et al. (2009) have shown that disparity gradients influence slant perception up to distances of at least 9 m. However, slant around a vertical axis on the basis of disparity gradients alone is actually problematic even at relatively close distances. It is usually underestimated and takes time to resolve (Gillam et al., 1984, 1988; van Ee and Erkelens, 1996). This has been attributed to its ambiguity with respect to azimuth (eccentricity relative to the median plane) (Mitchison and Westheimer, 1990; Gillam, 1993). A frontal plane surface centered on the median plane will produce the same disparity gradient as a slanted surface to the side of the median plane. This ambiguity may lead the visual system to rely on other slant information, or it may be disambiguated by information associated with variations in azimuth such as vertical disparity (Mayhew and Longuet-Higgins, 1982; Gillam and Lawergren, 1983). However, there has also been a suggestion that the slant of a surface around a vertical axis may be disambiguated by comparing the angular deviation of its disparity gradient from the horopter to its deviation from the normal to the line of sight on the basis of perspective information (Gillam, 1993). The important insight relevant to the present analysis is that the horopter, the frontal plane and the normal coincide in central vision but not in eccentric vision (see Figure 1B). Thus, information that a surface is on the horopter (i.e., has no horizontal disparity) does not necessarily mean that it has no slant relative to either the frontal plane or the normal, which in central vision would produce luminance differences in the two eyes. Thus, it is quite possible for a slanted surface to have both a luminance difference (reflecting a slant relative to the frontal plane or the normal) and to have zero disparity. This would only occur for an eccentric surface, but resolving information in this way would not necessarily lead to the *perception* that the surface is eccentric. It is already known that the stereo azimuth cue of vertical disparity that results in processing a surface as if it were eccentric (for example in the induced effect) does not result in seeing the surface as off to the side (Banks et al., 2002).

Although shape from shading is a well-known depth cue and is often considered together with disparity in cue integration models, the idea of shading disparity is different and rarely considered. Bülthoff and Mallott (1988) are some of the few to consider its role. They found that the presence of shading disparity as opposed to shape from shading (shading identical in the two eyes) added significantly to the perceived depth of ellipsoid shapes. Even though this situation is very different from the venetian blind effect it does show in a different context that a mechanism exists for relating binocular luminance differences and depth.

We have discussed two ways in which luminance differences could underlie the slant perceived in the venetian blind stimulus. Firstly, luminance information consistent with slant could conflict with and dominate ambiguous disparity information. Alternatively, the luminance difference could disambiguate the slant by causing processing of the surface as if it were in an eccentric position where a zero disparity surface could be slanted in a direction consistent with a luminance difference between the two eye's views.

It is obvious from looking at the line stereograms of Wheatstone (1838), with which he first demonstrated stereopsis, that he saw stereopsis not as a response to horizontal disparity alone but as an ability to interpret the differences in the perspective views of the two eyes of objects in depth. His drawings included horizontal disparity, but also orientation curvature, spatial frequency disparity, vertical disparity, and monocular regions, all of which have been studied in recent years especially since the application of a mid-level approach to the study of binocular vision. The venetian blind effect suggests that surface luminance disparity may also play a role in depth perception.
